# Public domain small-area cancer incidence data for New York State, 2005-2009

**DOI:** 10.4081/gh.2016.304

**Published:** 2016-04-18

**Authors:** Francis P. Boscoe, Thomas O. Talbot, Martin Kulldorff

**Affiliations:** 1 New York State Cancer Registry, New York Stat Department of Health, Albany, NY; 2 Bureau of Environmental & Occupational Epidemiology, New York State Department of Health, Albany, NY; 3 Harvard Medical School and Harvard Pilgrim Health Care Institute, Department of Population Medicine, Bostan, MA, USA

**Keywords:** Cancer incidence, Cancer mapping, Open data, Small-area

## Abstract

There has long been a demand for cancer incidence data at a fine geographic resolution for use in etiologic hypothesis generation and testing, methodological evaluation and teaching. In this paper we describe a public domain dataset containing data for 23 anatomic sites of cancer diagnosed in New York State, USA between 2005 and 2009 at the census block group level. The dataset includes 524,503 tumours distributed across 13,823 block groups with an average population of about 1400. In addition, the data have been linked with race/ethnicity and with socioeconomic indicators such as income, educational attainment and language proficiency. We demonstrate the application of the dataset by confirming two well-established relationships: that between breast cancer and median household income and that between stomach cancer and Asian race. We foresee that this dataset will serve as the basis for a wide range of spatial analyses and as a benchmark for evaluating spatial methods in the future.

## Introduction

There has long been a demand for a free high-quality publicly available dataset of cancer incidence at a fine geographic resolution. Such a dataset would provide a common reference that researchers could use for examining the potential relevance of etiologic risk factors, for evaluating and comparing spatial statistical methods and for pedagogic purposes. Within the United States, researchers have made extensive use of a dataset consisting of 592 cases of leukaemia diagnosed in central New York between 1978 and 1982 that was originally described by Waller *et al.* (1992, 1994). Despite its age, this dataset continues to be cited frequently (Gangnon, 2012; Rogerson, 2012). Alternatively, some researchers make use of study-specific datasets that cannot be shared for reasons of patient confidentiality, while others rely on synthetic data (Guo and Wang, 2011).

The New York State Department of Health (NYSDOH) has published a public domain dataset that goes a long way toward meeting this demand (NYSDOH, 2013a). It was the result of state legislation passed in 2010 mandating that NYSDOH make detailed cancer data available to the public (State of New York, 2013). The dataset consists of observed and expected counts for 23 anatomic sites of cancer at the neighbourhood scale, diagnosed between 2005 and 2009. An enhanced version of the data linked to United States census data is also available at http://tinyurl.com/onpd6zp. In this paper, we describe this dataset, create some basic cancer surveillance maps, and conduct some basic ecologic analyses. For the latter, we examine the associations between female breast cancer and median household income and between stomach cancer incidence and the proportion of Asians in the population. These examples were selected because the relationships are well established and should presumably be evident in the data. Additionally, the fine geographic resolution of these data should allow for additional insights beyond what can be obtained through conventional non-spatial analysis.

Regarding the ecologic analyses, stomach cancer rates among Asian-Americans are consistently high with infection by *Helicobacter pylori* regarded as the primary etiologic factor (Fock and Ang, 2010). Stomach cancer rates among Asians are more than double those for non-Hispanic whites both in New York State (New York State Cancer Registry, 2014) and in the eighteen states and metropolitan areas included in the National Institutes of Health's Surveillance, Surveillance, Epidemiology and End Results (SEER) programme (National Cancer Institute, 2012). Minor risks for stomach cancer not specific to Asians include a high intake of salt-preserved foods and dietary nitrite combined with low intake of fruit and vegetables, along with smoking (Brenner *et al*., 2009). Stomach cancer also exhibits large gender differences with rates among men roughly double those in women.

Breast cancer incidence has consistently been found to relate to higher socioeconomic status (SES), as described in a recent review of 90 studies over the 1978-2009 period by Klassen and Smith (2011). Most researchers have used SES as a marker for specific behaviours or exposures known or believed to have an etiologic relationship with cancer, including age at menarche and menopause, parity, nulliparity, age at first birth and oral contraceptive use. However, since many of these behaviours tend to occur in combination as part of broader lifestyle patterns, the reviewers conclude that a compelling case can be made for considering SES as a direct risk factor for breast cancer (Klassen and Smith, 2011).

## Materials and Methods

### Cancer incidence data

The data represent all invasive malignant tumours diagnosed among New York State residents between 2004 and 2009 and recorded in the New York State Cancer Registry (NYSCR) as of November 3, 2011. The NYSCR achieved the highest national certification levels for data timeliness, completeness and quality over this entire time period (North American Association of Central Cancer Registries, 2014). The 23 anatomic sites included in the dataset account for 86 percent of all reportable malignant tumours in New York State. This set of cancer sites encompasses the most common cancers plus certain less common ones with well-hypothesised environmental or occupational aetiologies. A listing of the 23 sites and the number of tumours diagnosed in the 2005-2009 period is given in Table 1. The dataset additionally includes counts for all other sites combined (71,785), for a grand total of 524,503 tumours. Only 503 tumours (0.1%) had to be excluded because they lacked age, sex and/or any address information. The full dataset can be freely downloaded from the NYSDOH web site at https://health.data.ny.gov/Health/Cancer-Mapping-Data-2005-2009/cw3n-fkji.

The data are provided at the level of the census block group. Block groups are relatively homogeneous statistical units of about 600 to 3000 people and are the smallest unit for which sample-based data are tabulated by the United States Census Bureau's American Community Survey (United States Census Bureau, 2014). In the 2010 US Census, there were 15,464 block groups in New York State, with an average population of 1253; 94% of these had a population of between 600 and 3000. In order to protect patient confidentiality, a block group needed to have a minimum of 6 tumours diagnosed among males and 6 tumours diagnosed among females, summed over all cancer sites, for it to have been included in the dataset. Block groups not meeting this requirement were merged with neighbouring block groups from the same census tract using a downloadable geographic aggregation tool developed in SAS, version 9.2 (SAS Institute, Inc., Cary, NC, USA) (Babcock, 2010; Talbot and LaSelva, 2010). This resulted in a reduction in the number of block groups by 11% to 13,823 and a corresponding increase in average population to 1,402. In the dataset, merged block groups are identified with a custom code containing the letters DOH so as not to be confused with census codes. The census block groups comprising each merged block group are provided in a separate crosswalk file.

For approximately 94% of the cancer cases, the block group was determined by automated matching of the address at diagnosis to a street reference file maintained by the New York State Office of Cyber Security. For about 5% of the cases, the block group was determined by clerical review of the address where it failed to match the reference file for reasons such as misspelling, use of unofficial street name, post office box-only address, partial or ambiguous address, or address too new to have been included in the street reference file. The clerical review was performed by NYSCR staff using various online reference sources and the New York State Department of Motor Vehicles database. For the remaining 0.75% of the cases, the block group was imputed by randomly matching with a record sharing the same ZIP code. Expected counts were calculated using the indirect standardisation method, adjusted for sex and 5-year age groups up to 85+, using the 2010 census counts for New York State. As a consequence, for every cancer site, the sum of the expected counts equals the sum of the observed counts. In exchange for such fine geographic detail, other aspects of the data had to be omitted to ensure patient confidentiality. Accordingly, there is no information on the age, sex, race, ethnicity, or any other demographic characteristics of the cases, except where the sex is implied by the cancer site. These data can be obtained elsewhere on the NYSDOH web site at the county or state level.

### Cluster membership data

In addition to observed and expected counts, the public domain dataset also includes an indicator variable used to highlight block groups with unusually high or low cancer incidence, as determined using the spatial scan statistic (Kulldorff, 1997). A block group was defined as a high-incidence area if i) it was included in the most likely high-incident-rate cluster detected by the spatial scan statistic, provided the cluster was statistically significant at the alpha=0.05 level; or ii) it was included in a non-overlapping secondary cluster also statistically significant at the alpha=0.05 level; and iii) the observed rate was at least 50% higher than the expected rate. A block group was defined as a low incident area in the same manner, except here the expected rate had to have been at least 50% higher than the observed rate. The third criterion was imposed to minimise the tendency to identify small absolute differences in risk between large portions of the state (most typically between New York City and upstate New York) and instead emphasise smaller areas with greater variation in risk (Boscoe *et al.*, 2003). Each record in the dataset thus contains the block group identifier, observed counts, expected counts and cluster membership status (high, low, or neither) for each of the 23 cancer sites, plus all other sites combined.

### Enhanced data

The enhanced dataset adds the populations for each block group by sex, race, ethnicity, average household size, number of occupied and vacant housing units, number of persons above and below the poverty line, number of persons in each of 16 income categories, median household income, number of persons with and without at least a high-school education and number of persons with limited English proficiency. The population counts here are from the 2010 United States Census Bureau (2011a) and the socioeconomic data are from the 2006-2010 American Community Survey (United States Census Bureau, 2011b).

### Interactive cancer incidence map

**Figure 1:**
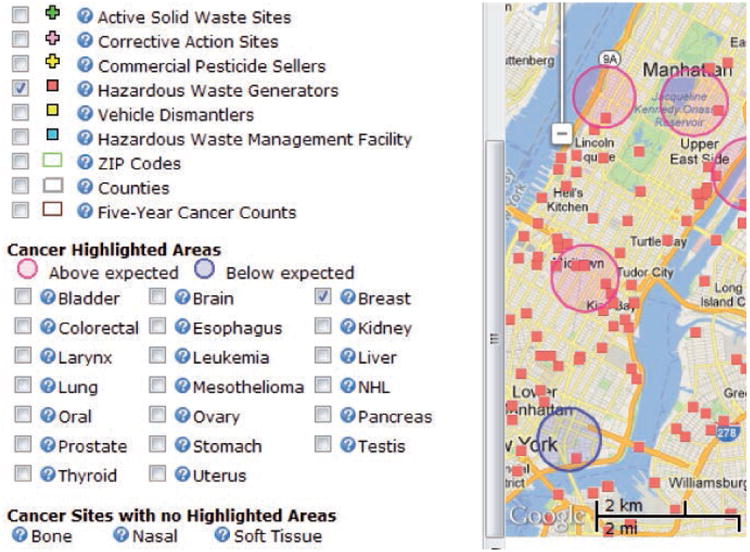
Representative screen capture from the Environmental Facilities and Cancer Mapping web site (https://apps.health.ny.gov/statistics/cancer/environmental_facilities/mapping/map/).

In addition to the downloadable data, an interactive map of the data can be viewed on the NYSDOH web site (2013b). This map also allows the viewing of locations of regulated environmental sites and facilities within the state, ranging from fuel tanks to commercial pesticide sellers to hazardous waste sites. Figure 1 shows a screen capture from this site, showing breast cancer and hazardous waste generators in a portion of Manhattan. The web site is designed for viewing on a typical computer screen with a horizontal format, so the map actually extends much further to the right.

### Descriptive maps

For this paper, we constructed two data maps, the first of which shows standardised incidence ratios (SIRs, the ratio of observed to expected cases) for female breast cancer in Manhattan, while the second shows clusters for the same data using the cluster membership indicator variable described above. We chose Manhattan because of its international familiarity and because areas much larger than this are difficult to depict on a standard page size. For the SIR map, we classified the SIRs into seven categories and used a diverging colour scheme centred on the state-wide rate (that is, centred on an SIR of 1). Note that the SIRs and clusters are relative to New York State and not to Manhattan or New York City.

### Ecological analyses

We assessed the relationship between stomach cancer and Asian race by calculating the percentage of the population in each block group with a race of *Asian alone* as measured in the 2010 Census by cluster membership (high, low, or neither). Additionally, we tabulated the relative risk (RR) of stomach cancer for different concentrations of Asians, independent of the cluster locations. For breast cancer, we used median household income data for the 2006-2010 period from the American Community Survey. We calculated the median household income by cluster membership, and then the RR of breast cancer for each income decile, independent of cluster location. All calculations were performed using SAS version 9.2 (SAS Institute).

## Results

### Descriptive maps

**Figure 2:**
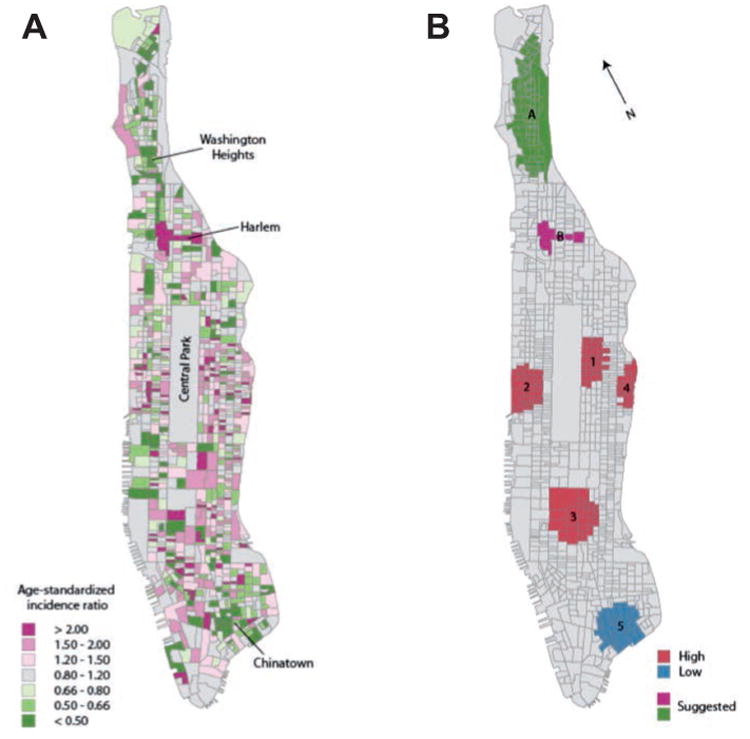
Breast cancer incidence, Manhattan, 2005-2009. Standardised incidence ratios (A) and by block group (B). Clusters as defined by the spatial scan statistic (red and blue) and other areas of interest suggested by the signal intensity ratio map (magenta and green).

Figure 2 shows the two views of the breast cancer data for Manhattan. The SIR map, containing values for 981 block groups, is noisy given that the mean number of breast cancer cases per block group is 6, with a range from 0 to 77. Even so, general patterns of high rates east and west of Central Park and low rates in Chinatown can be discerned. The cluster map identifies four areas of high rates and one area of low rates that are numbered on the map. Descriptive statistics for these cluster areas are given in Table 2. Cluster 4 also includes data from two block groups outside of Manhattan. Each of these five clusters meets the criteria described above: statistically significant, non-overlapping and with a RR greater than 1.5 or less than two-thirds.

Because interesting spatial patterns might not necessarily follow a circular shape, or because the no-overlap rule may cause interesting patterns on Manhattan to be masked by more pronounced patterns in nearby Brooklyn, Queens or Bronx, we attempted to use the SIR map to identify additional areas of interest from among those not included in a cluster. These are labelled A and B on the cluster map: a seemingly low area centred in Washington Heights and a seemingly high area in Harlem, both elliptical in shape. However, neither met all of the inclusion criteria. Atthough the Harlem cluster had more than a doubling of risk, the number of cases was insufficient to reach statistical significance. Conversely, the Washington Heights cluster was statistically significant, but the RR was above the cut-off used to identify low-risk clusters.

### Ecological analyses

The stratification of stomach cancer cases by cluster membership is shown in Table 3. Elevated clusters comprise 27.7% of the state population and 51.8% of the Asian population, meaning that Asians are 1.87 times more likely to reside in an elevated stomach cancer cluster than not. They are similarly less likely to live in a non-cluster area and much less likely to live in a low-cluster area. Stratification of stomach cancer cases by the percent Asian within each block group yields the results shown in Table 4. Majority-Asian block groups have a stomach cancer incidence rate that is more than double the average rate in the state. The rate is slightly elevated for block groups that are 10 to 50% Asian, and below the expected rate for block groups less than 10% Asian.

**Figure 3:**
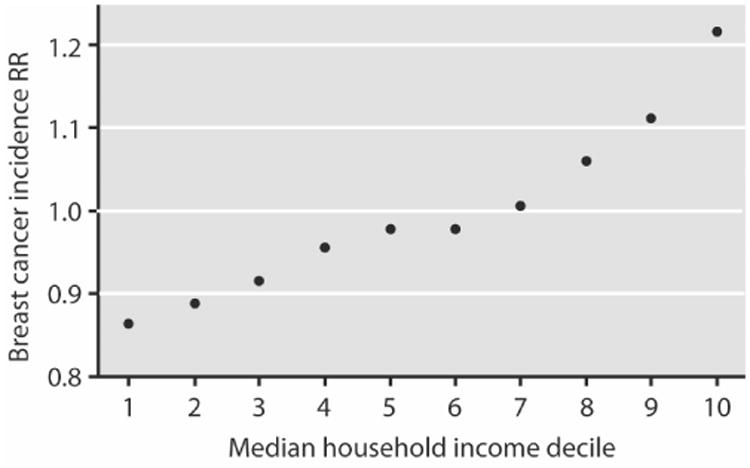
Relationship between median household income and breast cancer incidence in New York State at the block group level. Income figures based on 2010 census. Cancer incidence based on 2005-2009 diagnoses.

The stratification of breast cancer cases by cluster membership is given in Table 5. The difference in household median income between high-and low-cluster areas is a factor of 3. Stratifying the block groups by median income deciles shows a nearly monotonic increase in risk with increased income, including a 40% difference in risk between the highest and lowest deciles (Figure 3).

## Discussion

We have described a highly granular public-domain cancer incidence dataset and conducted some simple analyses using the data as published. Our intention was to demonstrate that this dataset is a convenient resource for the detailed exploration of spatial patterns of disease and for the evaluation and comparison of different spatial statistical and epidemiological methods.

We began by mapping SIRs and high and low clusters directly from the dataset itself (Figure 2). An SIR map is an obvious way of viewing the data, but it has limited utility when the number of counts per geographic unit is small. Using the eye to identify some potentially interesting patterns on this map did not identify any that were missed by the spatial scan statistic, though there still could be some potential public health relevance to the 39 cases identified in a small section of Harlem where only 18 were expected. We do note that SaTScan, the software for calculating the spatial scan statistic, has the capability of identifying elliptical clusters, but this feature was not used in the dataset described here and has only been used infrequently by researchers. Indeed, SaTScan has many customisable parameters (Amin *et al.*, 2014) and users of these data are not bound by the cluster memberships included in the file. The spatial scan statistic is just one technique for boosting the signal-to-noise ratio in small-area disease data and many other techniques are available (Talbot *et al.*, 2000; Johnson, 2004; Ozonoff *et al.*, 2005).

Next, we conducted two simple ecological analyses using the dataset. We identified a 2.5-fold increased risk between majority-Asian and non-Asian neighbourhoods, which is greater than the roughly 2-fold risk between Asians and non-Asians in both New York State and SEER. This result suggests that stomach cancer risk could be more acute in ethnic enclaves than among Asians generally. This in turn could be related to the nationalities that tend to live in such enclaves (for example, Chinese and Korean) as opposed to those who are more assimilated and dispersed (such as Indian and Filipino) and their respective risks of *H. pylori* infection. Alternatively, it could simply be because of geographical variation in risk among the Asian population (McCracken *et al.*, 2007). A natural follow-up question would be to investigate how stomach cancer rates among a particular group (say, Koreans) in Korean-majority neighbourhoods compare with those among Koreans in Korean-minority neighbourhoods. Such distinctions are seldom made in epidemiological studies but have implications for how doctors and public officials communicate risks to various racial and ethnic groups. Investigating this question is well beyond the scope of this paper and would require more detailed data than what is in the public dataset, but we mention it here because it is a good example of the kinds of novel hypotheses that can emerge when data are available at a fine geographic resolution.

Our second application focused on the relationship between breast cancer and socioeconomic status. SES remains an understudied dimension of public health, in part because these data are rarely collected directly by population-based surveillance systems (Toprani and Hadler, 2013). Our finding of more than a 40% difference in risk between the highest and lowest income deciles is consistent with previous findings (Klassen and Smith, 2011). Much of what is known about the relationship between breast cancer and SES has come from case-control and cohort studies; our analysis had the advantage of being relatively simple and straightforward to execute. As such, it lends itself to use in classroom teaching. Two of the authors of this paper have taught a graduate-level class in Geographic Information Systems (GIS) and Public Health for over a decade, and one of the greatest difficulties in the class has been to locate geographically rich datasets that allow interesting findings to be generated within a period of about four to six weeks (the time between when basic competency in GIS is achieved and the end of the semester). While it is possible to obtain interesting results from coarse datasets at the state or county level, the relationships of interest are often diluted by their highly heterogeneous nature. For example, New York has 62 counties but over half of the population lives in the seven which comprise New York City and Long Island. It makes no sense to assign identical socioeconomic characteristics to each person in Manhattan or Brooklyn. At the other extreme, a semester is too short to make use of datasets requiring outside permission. The dataset also lends itself to methodological applications. For example, we know of one doctoral student who is using these data to study the effects of the modifiable areal unit problem on health outcomes at varying spatial resolutions (Nelson, 2014). Two master's students at another institution used these data as the basis for their capstone projects (William Scheider, University of Buffalo Department of Epidemiology and Environmental Health, personal correspondence). It has also been used in training workshops on spatial statistical methods given at several national conferences. More broadly, the dataset has the potential to serve as a benchmark dataset for the evaluation and comparison of different spatial statistical methods involving spatial aggregation, clustering, smoothing and regression (Kulldorff *et al.*, 2004; Auchincloss *et al.*, 2012). Here we limited ourselves to analyses drawn directly from the data as published – the observed and expected counts, cluster memberships and linked census variables – but the applicability of these additional spatial statistical methods should be self-evident. Further, by including common as well as rare cancers, different aspects of the methods can be explored. The breadth of cancer sites also allows the findings to be interpreted in the context of known and hypothesised etiological relationships. In short, these data offer far more options than the 30-year old dataset of 592 leukaemia cases from central New York.

## Conclusions

Of course, no single dataset can serve all needs. Perhaps the largest limitation of these data is the lack of block group information on the ages or age ranges of the cancer patients. While the age structure of the population is captured by the expected case counts, it is not possible to use these data to do age-specific analysis (for example, clusters of cancer patients under age 65) or to apply a different population reference standard. Similarly, the lack of individual sex, race or ethnicity information constrains the kinds of analyses that can be performed, as does the absence of information on cancer stage, sub-site, histology, treatment or survival. While this is a drawback for some epidemiological investigations, it is typically not an issue when evaluating and comparing spatial statistical methods. In summary, we anticipate that this dataset can serve as a foundation for many methodological and epidemiological spatial analyses in the years ahead.

## Figures and Tables

**Table 1:** Cancer sites, international classification of diseases for oncology, and numbers of tumours diagnosed in the 2005-2009 period and included in the dataset.

Cancer site (ICD-O-3 classification according to Fritz *et al.,* 2000)	Tumours 2005-2009 (n)
Prostate (C61)	78,162
Female breast (C50)	72,296
Lung and bronchus (C34)	67,217
Colon and rectum (C18-C20, C26.0)	49,801
Bladder, including *in situ* (C67)	25,134
Non-Hodgkin lymphoma (morphologies 9590-9596, 9670-9671, 9673, 9675, 9678-9680, 9684, 9687, 9689-9691, 9695, 9698-9702, 9705, 9708-9709, 9714-9719, 9727-9729; 9823, 9827 except where site is C42.0, C42.1 or C42.4)	22,279
Uterus (C54-C55)	17,194
Kidney and renal pelvis (C64-C65)	16,371
Thyroid (C73)	15,109
Leukaemia (morphologies 9733, 9742, 9800-9949, 9963-9964)	14,091
Pancreas (C25)	13,927
Oral cavity and pharynx, excluding nasopharynx (C00-C10, C12-C14)	10,799
Stomach (C16)	9285
Liver and intrahepatic bile duct (C22)	8342
Ovary (C56)	7582
Brain and other nervous system (C70-C72)	6714
Oesophagus (C15)	5467
Larynx (C32)	4179
Soft tissue (C38.0, C47, C49)	3385
Testis (C62)	2690
Bone and joint (C40-C41)	1026
Mesothelioma (morphologies 9050-9055)	979
Nasal cavity and nasopharynx (C11, C30-C31)	689

ICD-O-3, International Classification of Diseases for Oncology (third edition).

**Table 2:** Statistics for breast cancer clusters on Manhattan.

Cluster	Block groups (n)	Observed (n)	Expected (n)	RR	Log-likelihood	P
1	36	330	206.1	1.60	31.5	<0.001
2	34	292	192.6	1.52	22.2	<0.001
3	23	150	88.6	1.69	17.6	<0.001
4	21°	211	139.7	1.51	15.7	0.003
5	38	167	265.4	0.63	21.1	<0.001
A	81	384	518.4	0.74	19.3	<0.001
B	4	39	18.0	2.17	9.2	0.120

RR, relative risk.

° Includes also one block group on Roosevelt Island and one block group in Queens, both to the east of Manhattan and not shown in Figure 2.

**Table 3:** Asian population by stomach cancer cluster type.

Geographic location	New York State population (%)	New York State Asian population (%)	Index of concentration (%)
High cluster	27.7	51.8	1.87
Not in a cluster	52.7	41.0	0.78
Low cluster	19.6	7.2	0.37

**Table 4:** Stomach cancer risk by Asian population proportion.

Asian percentage in block group (%)	Observed stomach cancers (n)	Expected stomach cancers (n)	RR
≥50	435	192	2.27
10-50	1877	1586	1.18
5-10	1347	1356	0.99
<5	5626	6150	0.91

RR, relative risk.

**Table 5:** Median household income by breast cancer cluster type.

Geographic location	New York State households (%)	Median household income, 2010 (US$)
High cluster	5.9	102,556
Not in a cluster	92.1	56,622
Low cluster	1.9	35,658
